# Pain in dementia

**DOI:** 10.1097/PR9.0000000000000803

**Published:** 2019-12-25

**Authors:** Wilco Achterberg, Stefan Lautenbacher, Bettina Husebo, Ane Erdal, Keela Herr

**Affiliations:** a Department of Public Health and Primary Care, Leiden University Medical Center, Leiden, the Netherlands; b Physiological Psychology, University of Bamberg, Bamberg, Germany; c Department of Global Public Health and Primary Care, Centre for Elderly and Nursing Home Medicine, Faculty of Medicine, University of Bergen, Bergen, Norway; d University of Iowa College of Nursing, Iowa City, IA, USA

**Keywords:** Pain, Pain assessment, Pain management, Dementia, Pharmacotherapy, Nonpharmacological interventions

## Abstract

The ageing revolution is changing the composition of our society with more people becoming very old with higher risks for developing both pain and dementia. Pain is normally signaled by verbal communication, which becomes more and more deteriorated in people with dementia. Thus, these individuals unnecessarily suffer from manageable but unrecognized pain. Pain assessment in patients with dementia is a challenging endeavor, with scientific advancements quickly developing. Pain assessment tools and protocols (mainly observational scales) have been incorporated into national and international guidelines of pain assessment in aged individuals. To effectively assess pain, interdisciplinary collaboration (nurses, physicians, psychologists, computer scientists, and engineers) is essential. Pain management in this vulnerable population is also preferably done in an interdisciplinary setting. Nonpharmacological management programs have been predominantly tested in younger populations without dementia. However, many of them are relatively safe, have proven their efficacy, and therefore deserve a first place in pain management programs. Paracetamol is a relatively safe and effective first-choice analgesic. There are many safety issues regarding nonsteroidal anti-inflammatory drugs, opioids, and adjuvant analgesics in dementia patients. It is therefore recommended to monitor both pain and potential side effects regularly. More research is necessary to provide better guidance for pain management in dementia.

**Key Points**

Pain in dementia is very prevalent and difficult to assess. Next to easy self-report measures, observational instruments are necessary in clinical practice.
Nonpharmacological management programs are the first line of choice, often in combination with analgesics.
Paracetamol is relatively safe in this population, and safety issues with other analgesics (eg, nonsteroidal anti-inflammatory drugs, opioids, and adjuvants) should be considered with careful risk/benefit analysis for each individual to achieve quality pain care.

## 1. Epidemiology

### 1.1. The demographic revolution puts pain in dementia in the spotlight

In the 20th century, the world population has seen an incredible growth in life expectancy. First, reduced mortality in the first years in life was responsible for this growth, but in the past decades, and in the coming decades, the growth is now impacted by many already “old” people (older than 60 years) getting “very old” (older than 80 years). Larger proportions of people in most societies will be relatively older and the absolute number of very old persons is rapidly growing around the globe.^66^ This also changes the spectrum of morbidity with many more age-related diseases, such as dementia, assuming a more prominent place in health care use, caregiver burden, and health care costs. Pain is also related to age and therefore the prevalence of pain and chronic pain is rising alongside the morbidity that it is associated with.^2,22,57^ Arthritis, in particular, is one of the diseases that is responsible for considerable pain in older persons.^78^

Unfortunately, the consequences of pain are more severe in older populations, especially on functional independence and social participation. The combination of a much higher prevalence with more severe consequences escalates pain in dementia to an important societal, clinical, and scientific challenge.

### 1.2. What do we know of pain in the different subtypes of dementia?

Dementia is a syndrome that can lead to confusion, memory loss, neuropsychiatric symptoms, and sometimes physical challenges. The Diagnostic and Statistic Manual of Mental Disorders Fifth Edition (DSM-5) does not mention dementia, but instead uses the term neurocognitive disorders, and classifies it as mild or major, on how severely the symptoms impact a person's ability to function independently in everyday activities.^5^

In a recent U.S. study, most patients with dementia were diagnosed with dementia not otherwise specified, with Alzheimer's disease (AD) being the most prevalent subtype.^31^ In the early stages, people with AD may find it hard to remember recent events, conversations, and names of people. In time, it becomes harder to communicate and judgment may become impaired. The person may feel disoriented and confused. Their behavior can change, and physical activities, such as swallowing and walking, will become harder. Vascular dementia (VD) is another highly prevalent cause of dementia, followed by Lewy body dementia and frontotemporal dementia (FTD).^31^ Often, people have mixed types of dementia, with both aspects of AD (such as amyloid plaques) and VD (white matter lesions). Also, other neurodegenerative diseases such as Parkinson disease and Huntington disease often are accompanied by dementia in the last stages of the disease. To know what the effects of pain are in different subtypes of dementia, several things have to be taken into account:

(1) The more general problems that arise in most type of dementias, such as difficulty in abstract thinking and verbal communication, and
(2) the location of the neuropathology.

The more general problems with abstract thinking and communication will be discussed in the assessment of pain in dementia. Here, we will discuss the specifics of different dementia subtypes related to the neuropathology, although we have only a few studies that look at pain in defined specific dementia populations.^11^

In AD, the classic neuropathological changes are predominantly in the temporal and parietal cerebral cortex and hippocampus. This neuropathology definitely is affecting several pain centers in the brain. Earlier work postulated that people with AD therefore, although they would be able to sense pain, could be less emotionally affected by it.^58^ However, several experimental studies have shown that it is unlikely that this is the case: both pain reflex studies and fMRI studies after pain stimulus show that the reaction to pain of patients with AD is even more pronounced.^20^

Vascular dementia has been associated with slightly higher pain prevalences, probably because of the possibility that the brain white matter lesions are the cause of central pain.^59^ One of the most recent studies on pain in VD suggests that people with VD have a similar pain intensity as cognitively intact persons, but seem to suffer more from it.^60^

Frontotemporal dementia and pain has only been investigated in 2 studies, one of them being experimental. Based on these studies, we assume that it is possible that patients with FTD have an increase in pain threshold, and possibly also in pain tolerance.^9^ Thus, different dementia types may experience pain differently. However, in the 2 most frequent forms of dementia, namely AD and VD, there is no indication for a reduced but instead for an augmented vulnerability to pain.

### 1.3. Prevalence of pain in dementia

In the community, more than half of the patients with dementia experience daily pain.^8^ In nursing homes around 60% to 80% of people with dementia regularly experience pain.^17^ There are many different causes, such as musculoskeletal, gastrointestinal, and cardiac conditions, but also genitourinary infections and wounds, such as pressure ulcers.^17^ Not all patients experiencing pain have chronic pain, but those who have are more likely to have an accelerated memory decline.^52^ Approximately 1 in 3 residents have moderate to severe pain, and patients with more severe dementia experience more pain than those with less severe dementia.^73^ In a recent study, nociceptive pain has been found as the most prominent type (70%) in a nursing home population, followed by a mix of nociceptive and neuropathic pain (25%).^73^

Of particular interest is orofacial pain, which is related to poor oral health care. It is prevalent in around 10% of the patients with dementia and may cause significant suffering.^70^

### 1.4. Consequences of pain in dementia

There is some evidence that pain in dementia is related to a variety of behavioural symptoms, such as depression, verbal abuse, wandering, agitation, and aggression.^67^ The relation of pain with functional impairment has not been studied so well in the dementia population, or studies were of low methodological quality. The one that was of relatively good quality showed a relation between pain and functional impairment.^45^

## 2. Pain assessment in patients with dementia

Competent pain assessment is a necessary prerequisite for good pain management and ideally considers several pain dimensions, namely intensity, location, affect, cognition, behavior, and social accompaniments. In case of patients with dementia, many cognitive and linguistic barriers prevent individuals from focusing on all these aspects. Those responsible for pain management must be adequately informed at the least about the presence and intensity of pain. Thus, limited and one-sided pain assessment is almost the rule in individuals with dementia, leading to deleterious consequences for their pain treatment or lack thereof.^12^ The best-possible forms of pain assessment will be briefly reviewed in the next paragraphs.

### 2.1. Self-report

The gold standard in pain assessment is the self-report either in less standardized forms as asked in interviews or in more standardized forms as requested in pain scales.^50^ Although it lies in the nature of the disease “dementia” that the capacity of self-monitoring inner states and reflecting them appropriately in self-report statements deteriorates over time and finally gets lost, self-report of pain should not be disregarded too early but still sought in mild to moderate forms of dementia. Referring to the Mini Mental State Examination,^29^ a cutoff score of 18 was suggested to divide individuals into those still able and those no longer able to self-report pain.^15^ However, it is necessary to adapt the form of self-report to the individual capabilities of the patient. The frequently used visual analogue scale, which requests the matching of a line length to the experienced intensity of pain, is far beyond the cognitive level of most patients even in early stages of dementia. Simple numerical or verbal scales and—later in the course of dementia—even simpler categorical questions (to be answered with “yes” or “no”) should be used. However, even minimal knowledge in the patients about the requests associated with answering certain simple scales does not always guarantee valid self-report of pain. Therefore, from a certain degree of cognitive and linguistic impairment on, it is advised to add an observer tool to self-report assessment, which takes on the leading role more and more in later stages of dementia. Thus, besides direct pain testing, neuropsychological screening of the cognitive status should become routine to become sensitive for the transition from possible to invalid self-report.

### 2.2. Observer ratings

There is general agreement that observer ratings of pain organized in short scales are necessary in moderate and severe forms of dementia to get valid and reliable information about the presence and intensity of pain. In addition, there is also wide agreement that 3 behavioral domains mirror pain-related states, which are namely facial responses, vocalization, and body movement or body posture.^75^ There are meanwhile numerous observer-rating scales available based on this principal, which, however, disagree substantially in the selection of the exact items for the 3 domains. A survey of the available scales is given in Table 1.

**Table 1:**
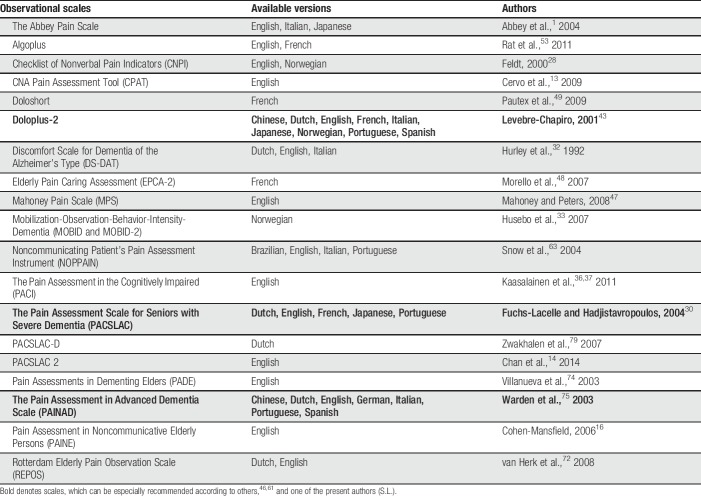
Survey of the most frequently used observational scales according to Zwakhalen et al.^80^ (2017; modified form).

The problems associated with these scales include poor or unproven reliability, lack of evidence for validity, and untested sensitivity of change. Also, implementation in practice is poor. However, even if the optimal tool has not yet been developed, it has turned out that as soon as any attempt of systematic pain assessment by such scales was implemented in nursing homes, pain management improved.^54^ Thus, it will be important to convince the end-users (mainly geriatric nurses) to apply such scales and help to further improve their usability.

The imperfect state of development of most observer-rating scales has inspired an American and a European research group to develop meta-tools out of the existing observer-rating scales, which try to make use of only the best possible items available. These attempts have become obtainable in 2 scales, namely Pain Intensity Measure for Persons with Dementia (available in English^27^) and PAIC-15 (Pain Assessment in Impaired Cognition; available in English and 8 other languages^18,21,41,68^) and await further testing.

A special challenge is pain assessment in end-of-life care, which requires special instruments with more focus on psychological distress. A few instruments are yet become available.^71^

### 2.3. Experimental methods

Experimental methods such as pain psychophysics (eg, pain threshold and tolerance threshold), brain imaging, neurophysiologic recordings (eg, SSEP and R-III reflex), and facial response coding are not easily used in the clinical context for pain assessment and are mainly reserved for research on individuals with dementia.^39^ Their great relevance is the demonstration of changes in nociception and pain processing associated with various forms of dementia (see paragraph “what do we know of pain in the different subtypes of dementia?” in this article). Thus, we now also know that the brain changes associated with dementia do not reduce pain to a degree, which make further attempts of pain management unnecessary. By contrast, most forms of dementia are even associated with enhanced nociception and pain processing.

### 2.4. Automatic pain recognition

The technical attempts of finding solutions to the problem of automatic pain recognition are meanwhile numerous and seem to be ideal for assisting pain measurement in noncommunicative (nonverbal) individuals.^19,40^ They are mainly video-based and target facial responses to pain but may add biosignals (eg, ECG, blood pressure, and EEG) and actigraphy. The momentarily available solutions may help to assess the immobile, bedridden patients under ideal conditions of illumination and no visual overlap, who show prototypical pain behavior without masking by the expression of other emotions. Furthermore, the machine learning algorithms applied for pain recognition are mainly trained on young individuals, which already let wrinkles invalidate this form of computer-driven pain diagnostics.^7^ In other words, use in the uncontrolled clinical environments of hospitals and care units in nursing homes is still out of reach.

For progress towards automatic pain recognition, interdisciplinary collaboration (nurses, physicians, psychologists, computer scientists, and engineers) is mandatory.

## 3. New developments in nonpharmacological management

Complexities of pain in older persons with dementia necessitate a comprehensive pain management approach that encompasses more than pharmacotherapy. For years, clinical practice guidelines have recommended incorporation of nonpharmacologic approaches as part of the pain management plan for older adults,^2^ but recent concern related to opioid use for chronic pain has increased attention to the effective use of nondrug approaches.^65^ Incorporation of nondrug techniques involves careful consideration of the unique patient circumstances, patient preferences, and evidence of effectiveness and guidance for selection of these interventions in the frail older person with dementia. Although evidence is growing, the majority of evidence for nondrug pain interventions has been conducted in cognitively intact older adults because those with dementia are typically excluded from most randomized control trials (RCTs). Evidence is accumulating on nondrug interventions to manage behavioral and psychological symptoms of dementia (BPSD), but few studies focus specifically on pain as the outcome of interest.

Exercise has been shown to be an effective nondrug intervention for pain in older adults; thus, it is reasonable to assume that it may be beneficial for pain in those with dementia.^57^ Choice of an exercise intervention, however, should take into consideration the individual's physical and cognitive status, health conditions, risk of falls, level of fitness, prior and current physical activity, support for implementation, and environmental factors. Approaches need to be tailored to the individuals understanding and abilities, capitalizing on significant other support/assistance with reasonable and achievable goals (Table 2).

**Table 2:**
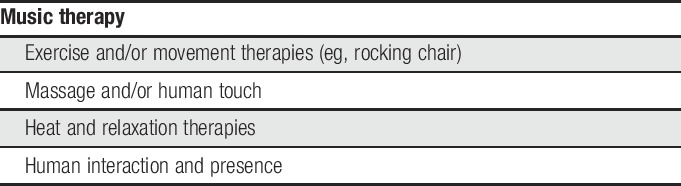
Most promising nonpharmacologic interventions for use in dementia.

Psychological interventions have a strong evidence base in adult samples, and growing evidence in older adults;^10^ however, research is sparse on use in dementia. Impairments in memory, language, executive function, visuospatial skill, and other processes impact the ability to effectively engage in these pain interventions, such as cognitive-behavioral therapy, mindfulness approaches (including biofeedback and relaxation training), and self-regulatory approaches (including biofeedback, relaxation training, and hypnotherapy). It is likely that these interventions are not feasible in those with moderate and severe dementia.

Recent reviews of nonpharmacologic interventions for the treatment of BPSD, including agitation and disruptive behavior that have both been associated with pain, provide some guidance. In a review of systematic reviews evaluating RCTs, Dyer et al.^23^ note that although quality of evidence for nonpharmacological interventions is low, lack of adverse events supports trialing techniques with potential benefit. Functional analysis-based interventions, such as behavior management and music therapy, demonstrated statistically significant improvements in BPSD. A second review of reviews conducted by Legere et al.^44^ on nonpharmacological approaches for BPSD not limited to RCTs concluded that there continues to be sparse high-quality evidence, with the strongest support for music therapy.

Pieper et al.^51^ found that behavioral interventions targeting pain, including music therapy, cognitive behavioral therapy, reflexology, Reiki, person-centered bathing or showering, and rocking chair therapy, were effective in reducing pain and behavioral symptoms in dementia. A recent integrative review examined the state of science on nonpharmacological intervention use for pain for older adults in long-term care facilities, many of which have dementia.^62^ Exercise, massage, heat therapy, and relaxation/rest were identified as significant interventions for persistent noncancer pain. Finally, a recent review of RCTs focused on complementary and alternative interventions to treat pain and agitation in dementia found massage, touch, and human interaction and presence are effective in reducing pain and agitation.^6^ Although evidence for efficacy is limited in this setting, incorporation of strategies that caregivers identify as potentially useful for the older person with dementia can be encouraged.

It is difficult to know whether any of the nonpharmacologic pain interventions are superior to another or when and how their use should be tailored to the individual's unique needs and characteristics. Studies are needed that use strong designs, include valid and reliable pain behavior outcomes, examine impact of dose of intervention, and establish feasibility, applicability, and cost-effectiveness for use in the long-term care setting.

## 4. New developments in pharmacological management

When it is not possible to relieve pain by either targeting the cause of pain and/or applying nonpharmacological strategies alone, pharmacological treatment of pain is necessary and remains a cornerstone in the care for people with dementia. The overall use of analgesics in nursing home patients is increasing worldwide.^42^ Dementia is common among nursing home patients, and despite concern that pain may be underdiagnosed in people with dementia, several studies have found that in more recent cohorts, nursing home patients with dementia were no less likely to receive analgesics compared to those without.^35,56^ However, these findings were predominantly from Scandinavian countries.

### 4.1. Paracetamol

Paracetamol/acetaminophen remains the mainstay for treating mild-to-moderate pain in advanced dementia, as reflected by 2 of the publications with most recent data reporting that 48% (2011; Norway) and 71% (2014; Australia) of nursing home patients with dementia receive paracetamol.^26,56,64,^ However, these figures are not directly comparable because analgesic use was defined as regular prescriptions only in the 2011 cohort, whereas the 2014 cohort included any use during the last 24 hours. Although limited, the available evidence suggests that paracetamol is effective and safe, and therefore represents an appropriate first choice for analgesic treatment in this population.^26,34,64^ It should be noted that although long-term use of paracetamol is common in people with dementia, no study has investigated the efficacy and safety of long-term treatment exceeding 3 months.^26,34,64^

### 4.2. Nonsteroidal anti-inflammatory drugs

Long-term use of nonsteroidal anti-inflammatory drugs (NSAIDs) is associated with increased risk of potentially serious adverse events, and should be avoided.^4,26^ Use of NSAIDs in people with dementia has decreased in recent years, and is generally low—in a sample of 169 nursing home patients with dementia in Australia in 2014, only 2.4% had received an NSAID during the last 24 hours.^56,64^ No study has investigated the risk and benefit of short-term use of NSAIDs for treating moderate-to-severe pain in people with dementia.^26^ The appropriate choice of NSAID, dose, and duration of use remains unclear and should be investigated further—it is possible that new evidence may place NSAIDs as an appropriate alternative to other agents with higher potential for cognitive and behavioral adverse effects, such as opioids, for treating acute pain of short duration in people with dementia.^26^

### 4.3. Opioids

Opioid analgesics are commonly prescribed for noncancer acute or chronic pain in people with dementia, and their use in this population has rapidly increased over the past decades.^35,55^ However, current developments highlight the need to investigate efficacy and safety in this population.^26^ Buprenorphine is a relatively new opioid analgesic with mixed activity and high potency. It has become increasingly prescribed to people with dementia because it is marketed as an easily administered transdermal patch formulation at low equianalgesic dose levels.^35,56^ The first double-blinded trial of buprenorphine in people with advanced dementia found high risk of adverse events and the adverse symptoms that were described overlapped with common BPSD in dementia such as changes in personality, confusion, sedation, or somnolence.^25^ This suggests that people with dementia may experience unexpected adverse symptoms that may go unnoticed in this population. Oxycodone and morphine are the only other opioids that have been tested in randomized controlled trials including people with dementia, and all trials were limited by a low number of participants.^26,34^ Although the results must be interpreted with caution due to lack of comparability between trials, the available evidence suggests that all treatments seem to reduce pain, and that oxycodone and morphine may be better tolerated.^34,55^ However, the relative efficacy and tolerability of different opioids in people with dementia and pain must be investigated further to establish evidence-based treatment guidelines and minimize the risk of harm while promoting pain relief.

### 4.4. Adjuvant analgesics

No clinical trials have investigated the safety and efficacy of adjuvant analgesics such as antidepressants and antiepileptic drugs for treating pain in dementia.^34,55^ People with dementia may be at increased risk of adverse events of psychotropic drugs, as well as drug–drug and drug–disease interactions. The risk–benefit relationship may therefore be skewed in this population, and studies of adjuvant analgesics for treating pain are needed to make evidence-based treatment decisions in this population.

### 4.5. Unresolved issues

Several important unresolved issues remain in relation to current guidelines and practice for the use of analgesic drugs in people with dementia. No evidence-based guidelines exist for treating pain in people with dementia; instead, general guidelines for the geriatric population are applied to this group despite lack of evidence for efficacy and safety in people with dementia. Clinical practice for pain management in people with advanced dementia also varies widely both within and across nations. Although several studies show that the overall use of analgesics is increasing, we do not know whether the right patients receive appropriate analgesic treatment in the correct dose. Recent studies show that not all those who receive analgesics have pain; similarly, many still have pain despite receiving analgesic treatment.^24^ The most recent data from 2014 report that within a Norwegian cohort of 407 nursing home residents with advanced dementia, 54.1% used paracetamol and 32.7% used opioids on a daily basis.^69^ On average, those who received analgesics had more pain compared to those who did not—however, in the former group, 46% were still registered as having mild or no pain.^69^ Most studies on medication use in dementia are cross-sectional and cannot determine whether the administered treatment is appropriate and had the intended effect—for those who still have pain, we do not know whether treatment has reduced pain intensity or not. Similarly, for those with no registered pain, we do not know whether they have been successfully treated, or whether treatment is even indicated. Therefore, a trend towards increased prescribing of analgesics in people with dementia does not equate improved quality of care.^3^ Longitudinal data, with assessments before and after treatment, and randomized pain intervention studies in particular are needed to evaluate the current quality of care and shed light on potential areas of improvement.

### 4.6. Interdisciplinary pain management and implementation in long term care

As with any patient with persistent pain, development of a comprehensive treatment plan is essential and, in the population of dementia, in particular, an interdisciplinary approach is key to establishment of a multimodal pain management plan. An interdisciplinary approach includes comprehensive assessment, managing polypharmacy and pharmacotherapy, psychological evaluation and support, physical rehabilitation, and interventions and interventional procedures.^38^ Before institutionalization, the team includes the person with dementia and their family caregiver to assure a patient-centered approach that incorporates the values, preferences, and needs of the person with dementia.^76^ When institutionalized, expertise in geriatric syndromes and conditions, knowledge of the complexities of pharmacotherapy and comorbidities, skill in recognizing, assessing and monitoring pain in those unable to self-report, and understanding of the long-term care environment and its impact on the older person with dementia are needed.

Organizations invested in an interdisciplinary team approach to managing the problems associated with dementia are best prepared to gather information that informs the treatment plan and engages individuals most likely to be effective in implementing the pain management plan. Challenges of creating a functional interdisciplinary team need to be addressed by the organization to promote use of nondrug therapies and establish reimbursement for multiple providers and nondrug therapies for pain management.^77^ Organizational policies and procedures are necessary that establish the approach to pain assessment in dementia and evidence-based pain management strategies, as well as resources for staff education and implementation of nonpharmacological interventions.

## 5. Conclusion

Pain is a challenge for persons with dementia, their loved ones, health care professionals, and society. Although in the past decade better assessment procedures including observational pain instruments have been developed and studied, implementation in practice is still disappointing. Good pain management is unfortunately also not implemented, which this is partly due to a lack of good studies on both pharmacological and nonpharmacological management. To effectively assess and manage pain in this vulnerable group, interdisciplinary collaboration (nurses, physicians, psychologists, computer scientists, and engineers) is essential. This article provides the latest state of the literature on this topic.

## Disclosures

The authors have no conflicts of interest to declare.
